# Supplementary material for: Food-based indices for the assessment of nutritive value and environmental impact of meals and diets: A systematic review protocol
Source: PLoS One. 2024 Dec 20;19(12):e0315894. doi: 10.1371/journal.pone.0315894 (PMC11661603; doi:10.1371/journal.pone.0315894)
Supplement: S2 File — (DOCX) [file pone.0315894.s002.docx]

**Supplementary Material File #2**

Food-based indices assessing nutritive value and environmental impact of meals and diets: A systematic review protocol.

Contents

[Appendix S1: Search String building: key word grouping from identified relevant papers 2](#_Toc169094403)

[Appendix S2: Search strategies **on April 12^th^ 2024 to April 17^th^ 2024** 4](#_Toc169094404)

[Table 1.1 Database: CAB Abstracts <1973 to present> 4](#_Toc169094405)

[Table 1.2. Database: Ovid Embase <1974 to present> 5](#_Toc169094406)

[Table 1.3 Database: FSTA <1969 to present> 6](#_Toc169094407)

[Table 1.4. Database: Ovid MEDLINE(R) <1946 to present> 7](#_Toc169094408)

[Table 1.5 Database: Science Citation Index & Social Citation Index <1945 to present> 8](#_Toc169094409)

[Table 1.6 Database: Scopus <1996 to present> 9](#_Toc169094410)

# Appendix S1: Search String building: key word grouping from identified relevant papers

| Key Themes | Key Words | | |
| --- | --- | --- | --- |
| Nutrition | - **Nutritional quality** - **Nutrients** - **Nutrition** - **Food** - Food Label - Food provision | - Energy - **Nutritional footprint** - Nutritionally invested environmental impact - **Nutrient rich food** - Plant-based protein-rich | - Nutrient density score - Nutrient density index - Nutritional profiling - Nutrient density - Nutritional adequacy |
| Environment / sustainability | - Environmental impacts reduction - **Environmental impact(s)** - Footprint - climate - Fossil energy use - Land occupation - Resource consumption - Freshwater eutrophication - Marine eutrophication - Global warming potential - Terrestrial acidification - Scarcity-weighted water use - Environmental indicators | - Sustainability - Sustainability indicators - Sustainability assessment - Exergy - **Climate change** - Climate impact - Environmental sustainability - Carbon dioxide equivalents - Carbon footprint - Water - Water footprint - Environment - Footprint - Resource productivity | - Life cycle assessment - Greenhouse gases - **Greenhouse-gas emissions** - Co2e emissions - Ecological impact - Water footprint - **Biodiversity** - **Land use / land-use** - LCA - Life cycle - **Life cycle assessment** - LCI |
| Behavior / health | - health impact - **Health** - **Food consumption** - **Consumption** - **Food choices** - Consumption | - Vegetarian - Sustainable eating - Food environment - Children health - Healthy citizen - Patterns | - Public health - Health indicators - Health score |
| Model | - Indicators - Muti-criteria analysis - **Framework** - **Impact(s)** | - Index - Validation - Tool - Model |  |
| Meal | - Canteen meal - Public school meals |  |  |
| Diet | - Dietary Assessment - Automated self-administered dietary assessment tool (ASA24) - 24-h dietary recall - National Health and Nutrition Examination Survey (NHANES) - Mobile food record | - **Dietary changes** - Mediterranean diet - **Diet** - **Sustainable diet** - **Diet cost** - Healthy diet - Diet quality | - Dietary shifts - Self-selected diets - Recommendations - Plant-based diets - Dietary patterns - New Nordic diet - Dietary guidelines |
| Other | - Institutional Food system sustainability - Food supply chain - Food waste management - Bioregional approach - Sustainable school programme - Data envelopment analysis - Choice - Food label | - Technology - Optimization - Mathematical programming - Linear programming - Linear programming modelling - Physical-activity - Patterns - Hotspots | - FOP-labelling - Consumer communication - Animal source food - Physical-activity questionnaire - Food security - Organic food - **Guidelines** - **Quality** - Diet-related greenhouse gas emission |

Words in bold are keywords that appeared more than once from identified relevant papers.

# Appendix S2: Search strategies **on April 12^th^ 2024 to April 17^th^ 2024**

## Table 1.1 Database: CAB Abstracts <1973 to present>

*Search strategy:*

| # ▲ | **Searches** | **Results** |
| --- | --- | --- |
| 1 | (nutri* NEAR/2 (quality or footprint or food* or profiling or density or score or index or adequacy)) or (food* NEAR/2 (label* or suppl* or consumption or choice or environment or pattern*)) or (eating NEAR/2 (indicator or score* or impact*)) or (feeding behavio$r* NEAR/2  (indicator or score* or impact*)) or (public health NEAR/2 (indicator or score* or impact*)) or (health* NEAR/2 (indicator or score* or impact*)) (Topic) | 377126 |
| 2 | (sustainab* NEAR/2 (impact or assess* or evaluat* or indicator? or health*)) or (environment* NEAR/2 (impact or assess* or evaluat* or indicator? or health*)) or (climate* NEAR/2 (impact or assess* or evaluat* or indicator? or health*)) or (land* NEAR/2 (clear* or "use*" or usage)) or (soil NEAR/2 (clear* or "use*" or usage)) or (water NEAR/2 ("use*" or usage or foot*)) or (life cycle NEAR/2 (evaluation or assessment)) or eutrophication or (carbon NEAR/2 (foot* or ecosystem)) or (fossil NEAR/2 fuels) (Topic) | 628541 |
| 3 | (model* or algorithm* or metric* or scor* or rank* or framework* or index or indices or tool* or inventor* or validation or "multi-criteria analys*" or guideline* or impact* or optim*) (Topic) | 7372892 |
| 4 | (meal* or menu* or recipe* or diet*) (Topic) | 810502 |
| 5 | 4 AND 3 AND 2 AND 1 | 5111 |
| 6 | animals or animal or mice or mus or mouse or murine or woodmouse or rats or rat or murinae or muridae or cottonrat or cottonrats or hamster or hamsters or cricetinae or rodentia or rodent or rodents or pigs or pig or swine or swines or piglets or piglet or boar or boars or sus scrofa or ferrets or ferret or polecat or polecats or “mustela putorius” or “guinea pigs” or “guinea pig” or cavia or callithrix or marmoset or marmosets or cebuella or hapale or octodon or chinchilla or chinchillas or gerbillinae or gerbil or gerbils or jird or jirds or merione or meriones or rabbits or rabbit or hares or hare or diptera or flies or fly or dipteral or drosphila or drosophilidae or cats or cat or carus or felis or nematoda or nematode or nematoda or nematode or nematodes or sipunculida or dogs or dog or canine or canines or canis or sheep or sheeps or mouflon or mouflons or ovis or goats or goat or capra or capras or rupicapra or chamois or haplorhini or monkey or monkeys or anthropoidea or anthropoids or saguinus or tamarin or tamarins or leontopithecus or hominidae or ape or apes or pan or paniscus or “pan paniscus” or bonobo or bonobos or troglodytes or pan troglodytes or gibbon or gibbons or siamang or siamangs or nomascus or symphalangus or chimpanzee or chimpanzees or prosimians or “bush baby” or prosimian or “bush babies” or galagos or galago or pongidae or gorilla or gorillas or pongo or pygmaeus or “pongo pygmaeus” or orangutans or pygmaeus or lemur or lemurs or lemuridae or horse or horses or pongo or equus or cow or calf or bull or chicken or chickens or gallus or quail or bird or birds or quails or poultry or poultries or fowl or fowls or reptile or reptilia or reptiles or snakes or snake or lizard or lizards or alligator or alligators or crocodile or crocodiles or turtle or turtles or amphibian or amphibians or amphibia or frog or frogs or bombina or salientia or toad or toads or “epidalea calamita” or salamander or salamanders or eel or eels or fish or fishes or pisces or catfish or catfishes or siluriformes or arius or heteropneustes or sheatfish or perch or perches or percidae or perca or trout or trouts or char or chars or salvelinus or “fathead minnow” or minnow or cyprinidae or carps or carp or zebrafish or zebrafishes or goldfish or goldfishes or guppy or guppies or chub or chubs or tinca or barbels or barbus or pimephales or promelas or “poecilia reticulata” or mullet or mullets or seahorse or seahorses or “mugil curema” or “atlantic cod” or shark or sharks or catshark or anguilla or salmonid or salmonids or whitefish or whitefishes or salmon or salmons or sole or solea or “sea lamprey” or lamprey or lampreys or pumpkinseed or sunfish or sunfishes or tilapia or tilapias or turbot or turbots or flatfish or flatfishes or sciuridae or squirrel or squirrels or chipmunk or chipmunks or suslik or susliks or vole or voles or lemming or lemmings or muskrat or muskrats or lemmus or otter or otters or marten or martens or martes or weasel or badger or badgers or ermine or mink or minks or sable or sables or gulo or gulos or wolverine or wolverines or minks or mustela or llama or llamas or alpaca or alpacas or camelid or camelids or guanaco or guanacos or chiroptera or chiropteras or bat or bats or fox or foxes or iguana or iguanas or “xenopus laevis” or parakeet or parakeets or parrot or parrots or donkey or donkeys or mule or mules or zebra or zebras or shrew or shrews or bison or bisons or buffalo or buffaloes or deer or deers or bear or bears or panda or pandas or “wild hog” or “wild boar” or fitchew or fitch or beaver or beav or jerboa or jerboas or capybara or capybaras (Topic) | 6048088 |
| 7 | 5 NOT 6 | 860 |
| 8 | 5 NOT 6 and 2024 or 2023 or 2022 or 2021 or 2020 or 2019 or 2018 or 2017 or 2016 or 2015 or 2014 or 2013 or 2012 or 2011 or 2010 or 2009 (Publication Years) | 842 |
| 9 | 8 and Journal Article (Document Types) | 723 |

## Table 1.2. Database: Ovid Embase <1974 to present>

*Search strategy:*

| # ▲ | **Searches** | **Results** |
| --- | --- | --- |
| 1 | (nutri* adj2 (quality or footprint or food* or profiling or density or score or index or adequacy)).ti,kw,ab. | 42597 |
| 2 | Food Labeling/ | 14250 |
| 3 | (food* adj2 label*).ti,kw,ab. | 3726 |
| 4 | exp Food Supply/ or food supply chain.ti,kw,ab. | 27613 |
| 5 | exp Eating/ or exp Feeding Behavior/ | 244796 |
| 6 | ((eating or feeding behavio?r* or public health or health*) adj2 (indicator or score* or impact*)).ti,kw,ab. | 82139 |
| 7 | (food* adj2 (consumption or choice or environment or pattern*)).ti,kw,ab. | 41577 |
| 8 | 1 or 2 or 3 or 4 or 5 or 6 or 7 | 421059 |
| 9 | carbon footprint/ or ecosystem/ or exp biodiversity/ | 500749 |
| 10 | Greenhouse Gases/ | 9128 |
| 11 | exp fossil fuels/ | 7436 |
| 12 | exp climate change/ or greenhouse effect/ | 78260 |
| 13 | exp soil pollutants/ | 17507 |
| 14 | waste water/ or exp water pollutants/ or water supply/ | 137658 |
| 15 | exp Forests/ | 45590 |
| 16 | ((sustainab* or environment*) adj2 (impact or assess* or evaluat* or indicator? or health*)).ti,kw,ab. | 94476 |
| 17 | (land* adj2 (clear* or "use*" or usage)).ti,kw,ab. | 29587 |
| 18 | (water adj2 ("use*" or usage or foot*)).ti,kw,ab. | 24377 |
| 19 | (life cycle adj2 (evaluation or assessment)).ti,kw,ab. | 6852 |
| 20 | eutrophication.ti,kw,ab. | 10472 |
| 21 | 9 or 10 or 11 or 12 or 13 or 14 or 15 or 16 or 17 or 18 or 19 or 20 | 865550 |
| 22 | (model* or algorithm* or metric* or scor* or rank* or framework* or index or indices or tool* or inventor* or validation or "multi-criteria analys*" or guideline* or impact* or optim*). ti,kw,ab. | 12420240 |
| 23 | Menu Planning/ or Meals/ or Cookbook/ or exp Diet/ | 651700 |
| 24 | (meal* or menu* or recipe* or diet*).ti,kw,ab. | 990017 |
| 25 | 23 or 24 | 1286536 |
| 26 | 8 and 21 and 22 and 25 | 3157 |
| 27 | exp animals/ not humans/ | 11753857 |
| 28 | 26 not 27 | 2027 |
| 29 | limit 28 to yr="2009 -Current" | 1872 |
| 30 | limit 29 to (article and journal) | 1121 |

## Table 1.3 Database: FSTA <1969 to present>

*Search strategy:*

| # ▲ | **Searches** | **Results** |
| --- | --- | --- |
| 1 | (nutri* NEAR/2 (quality or footprint or food* or profiling or density or score or index or adequacy)) or (food* NEAR/2 (label* or suppl* or consumption or choice or environment or pattern*)) or (eating NEAR/2 (indicator or score* or impact*)) or (feeding behavio$r* NEAR/2  (indicator or score* or impact*)) or (public health NEAR/2 (indicator or score* or impact*)) or (health* NEAR/2 (indicator or score* or impact*)) (Topic) | 115623 |
| 2 | (sustainab* NEAR/2 (impact or assess* or evaluat* or indicator? or health*)) or (environment* NEAR/2 (impact or assess* or evaluat* or indicator? or health*)) or (climate* NEAR/2 (impact or assess* or evaluat* or indicator? or health*)) or (land* NEAR/2 (clear* or "use*" or usage)) or (soil NEAR/2 (clear* or "use*" or usage)) or (water NEAR/2 ("use*" or usage or foot*)) or (life cycle NEAR/2 (evaluation or assessment)) or eutrophication or (carbon NEAR/2 (foot* or ecosystem)) or (fossil NEAR/2 fuels) (Topic) | 37622 |
| 3 | (model* or algorithm* or metric* or scor* or rank* or framework* or index or indices or tool* or inventor* or validation or "multi-criteria analys*" or guideline* or impact* or optim*) (Topic) | 635620 |
| 4 | (meal* or menu* or recipe* or diet*) (Topic) | 291078 |
| 5 | 4 AND 3 AND 2 AND 1 | 2112 |
| 6 | (animals or animal or mice or mus or mouse or murine or woodmouse or rats or rat or murinae or muridae or cottonrat or cottonrats or hamster or hamsters or cricetinae or rodentia or rodent or rodents or pigs or pig or swine or swines or piglets or piglet or boar or boars or sus scrofa or ferrets or ferret or polecat or polecats or “mustela putorius” or “guinea pigs” or “guinea pig” or cavia or callithrix or marmoset or marmosets or cebuella or hapale or octodon or chinchilla or chinchillas or gerbillinae or gerbil or gerbils or jird or jirds or merione or meriones or rabbits or rabbit or hares or hare or diptera or flies or fly or dipteral or drosphila or drosophilidae or cats or cat or carus or felis or nematoda or nematode or nematoda or nematode or nematodes or sipunculida or dogs or dog or canine or canines or canis or sheep or sheeps or mouflon or mouflons or ovis or goats or goat or capra or capras or rupicapra or chamois or haplorhini or monkey or monkeys or anthropoidea or anthropoids or saguinus or tamarin or tamarins or leontopithecus or hominidae or ape or apes or pan or paniscus or “pan paniscus” or bonobo or bonobos or troglodytes or pan troglodytes or gibbon or gibbons or siamang or siamangs or nomascus or symphalangus or chimpanzee or chimpanzees or prosimians or “bush baby” or prosimian or “bush babies” or galagos or galago or pongidae or gorilla or gorillas or pongo or pygmaeus or “pongo pygmaeus” or orangutans or pygmaeus or lemur or lemurs or lemuridae or horse or horses or pongo or equus or cow or calf or bull or chicken or chickens or gallus or quail or bird or birds or quails or poultry or poultries or fowl or fowls or reptile or reptilia or reptiles or snakes or snake or lizard or lizards or alligator or alligators or crocodile or crocodiles or turtle or turtles or amphibian or amphibians or amphibia or frog or frogs or bombina or salientia or toad or toads or “epidalea calamita” or salamander or salamanders or eel or eels or fish or fishes or pisces or catfish or catfishes or siluriformes or arius or heteropneustes or sheatfish or perch or perches or percidae or perca or trout or trouts or char or chars or salvelinus or “fathead minnow” or minnow or cyprinidae or carps or carp or zebrafish or zebrafishes or goldfish or goldfishes or guppy or guppies or chub or chubs or tinca or barbels or barbus or pimephales or promelas or “poecilia reticulata” or mullet or mullets or seahorse or seahorses or “mugil curema” or “atlantic cod” or shark or sharks or catshark or anguilla or salmonid or salmonids or whitefish or whitefishes or salmon or salmons or sole or solea or “sea lamprey” or lamprey or lampreys or pumpkinseed or sunfish or sunfishes or tilapia or tilapias or turbot or turbots or flatfish or flatfishes or sciuridae or squirrel or squirrels or chipmunk or chipmunks or suslik or susliks or vole or voles or lemming or lemmings or muskrat or muskrats or lemmus or otter or otters or marten or martens or martes or weasel or badger or badgers or ermine or mink or minks or sable or sables or gulo or gulos or wolverine or wolverines or minks or mustela or llama or llamas or alpaca or alpacas or camelid or camelids or guanaco or guanacos or chiroptera or chiropteras or bat or bats or fox or foxes or iguana or iguanas or “xenopus laevis” or parakeet or parakeets or parrot or parrots or donkey or donkeys or mule or mules or zebra or zebras or shrew or shrews or bison or bisons or buffalo or buffaloes or deer or deers or bear or bears or panda or pandas or “wild hog” or “wild boar” or fitchew or fitch or beaver or beav or jerboa or jerboas or capybara or capybaras) (Topic) | 444552 |
| 7 | 5 NOT 6 | 1410 |
| 8 | 5 NOT 6 and 2024 or 2023 or 2022 or 2021 or 2020 or 2019 or 2018 or 2017 or 2016 or 2015 or 2014 or 2013 or 2012 or 2011 or 2010 or 2009 (Publication Years) | 1405 |
| 9 | 8 and Journal Article (Document Types) | 1098 |

## Table 1.4. Database: Ovid MEDLINE(R) <1946 to present>

*Search Strategy:*

| # ▲ | **Searches** | **Results** |
| --- | --- | --- |
| 1 | (nutri* adj2 (quality or footprint or food* or profiling or density or score or index or adequacy)) .ti,kw,ab. | 34917 |
| 2 | Food Labeling/ | 4634 |
| 3 | (food* adj2 label*).ti,kw,ab. | 2729 |
| 4 | exp Food Supply/ or food supply chain.ti,kw,ab. | 18411 |
| 5 | exp Eating/ or exp Feeding Behavior/ | 265318 |
| 6 | ((eating or feeding behavio?r* or public health or health*) adj2 (indicator or score* or impact*)).ti,kw,ab. | 63150 |
| 7 | (food* adj2 (consumption or choice or environment or pattern*)).ti,kw,ab. | 33288 |
| 8 | 1 or 2 or 3 or 4 or 5 or 6 or 7 | 392776 |
| 9 | carbon footprint/ or ecosystem/ or exp biodiversity/ | 243091 |
| 10 | Greenhouse Gases/ | 2823 |
| 11 | exp fossil fuels/ | 31449 |
| 12 | exp climate change/ or greenhouse effect/ | 37954 |
| 13 | exp soil pollutants/ | 52379 |
| 14 | waste water/ or exp water pollutants/ or water supply/ | 200081 |
| 15 | exp Forests/ | 18024 |
| 16 | ((sustainab* or environment*) adj2 (impact or assess* or evaluat* or indicator? or health*)).ti,kw,ab. | 81053 |
| 17 | (land* adj2 (clear* or "use*" or usage)).ti,kw,ab. | 27416 |
| 18 | (water adj2 ("use*" or usage or foot*)).ti,kw,ab. | 21496 |
| 19 | (life cycle adj2 (evaluation or assessment)) .ti,kw,ab. | 4355 |
| 20 | eutrophication.ti,kw,ab. | 8408 |
| 21 | 9 or 10 or 11 or 12 or 13 or 14 or 15 or 16 or 17 or 18 or 19 or 20 | 631506 |
| 22 | (model* or algorithm* or metric* or scor* or rank* or framework* or index or indices or tool* or inventor* or validation or "multi-criteria analys*" or guideline* or impact* or optim*).ti,kw,ab. | 9440617 |
| 23 | Menu Planning/ or Meals/ or Cookbook/ or exp Diet/ | 341437 |
| 24 | (meal* or menu* or recipe* or diet*).ti,kw,ab. | 783154 |
| 25 | 23 or 24 | 894698 |
| 26 | 8 and 21 and 22 and 25 | 3121 |
| 27 | exp animals/ not humans/ | 5212238 |
| 28 | 26 not 27 | 2228 |
| 29 | limit 28 to yr="2009 -Current" | 2086 |
| 30 | limit 29 to journal article | 2054 |

## Table 1.5 Database: Science Citation Index & Social Citation Index <1945 to present>

*Search strategy:*

| # ▲ | **Searches** | **Results** |
| --- | --- | --- |
| 1 | (nutri* NEAR/2 (quality or footprint or food* or profiling or density or score or index or adequacy)) or (food* NEAR/2 (label* or suppl* or consumption or choice or environment or pattern*)) or (eating NEAR/2 (indicator or score* or impact*)) or (feeding behavio$r* NEAR/2  (indicator or score* or impact*)) or (public health NEAR/2 (indicator or score* or impact*)) or (health* NEAR/2 (indicator or score* or impact*)) (Topic) | 379496 |
| 2 | (sustainab* NEAR/2 (impact or assess* or evaluat* or indicator? or health*)) or (environment* NEAR/2 (impact or assess* or evaluat* or indicator? or health*)) or (climate* NEAR/2 (impact or assess* or evaluat* or indicator? or health*)) or (land* NEAR/2 (clear* or "use*" or usage)) or (soil NEAR/2 (clear* or "use*" or usage)) or (water NEAR/2 ("use*" or usage or foot*)) or (life cycle NEAR/2 (evaluation or assessment)) or eutrophication or (carbon NEAR/2 (foot* or ecosystem)) or (fossil NEAR/2 fuels) (Topic) | 889407 |
| 3 | (model* or algorithm* or metric* or scor* or rank* or framework* or index or indices or tool* or inventor* or validation or "multi-criteria analys*" or guideline* or impact* or optim*) (Topic) | 24359206 |
| 4 | (meal* or menu* or recipe* or diet*) (Topic) | 1152028 |
| 5 | 1 AND 2 AND 3 AND 4 | 4097 |
| 6 | (animals or animal or mice or mus or mouse or murine or woodmouse or rats or rat or murinae or muridae or cottonrat or cottonrats or hamster or hamsters or cricetinae or rodentia or rodent or rodents or pigs or pig or swine or swines or piglets or piglet or boar or boars or sus scrofa or ferrets or ferret or polecat or polecats or “mustela putorius” or “guinea pigs” or “guinea pig” or cavia or callithrix or marmoset or marmosets or cebuella or hapale or octodon or chinchilla or chinchillas or gerbillinae or gerbil or gerbils or jird or jirds or merione or meriones or rabbits or rabbit or hares or hare or diptera or flies or fly or dipteral or drosphila or drosophilidae or cats or cat or carus or felis or nematoda or nematode or nematoda or nematode or nematodes or sipunculida or dogs or dog or canine or canines or canis or sheep or sheeps or mouflon or mouflons or ovis or goats or goat or capra or capras or rupicapra or chamois or haplorhini or monkey or monkeys or anthropoidea or anthropoids or saguinus or tamarin or tamarins or leontopithecus or hominidae or ape or apes or pan or paniscus or “pan paniscus” or bonobo or bonobos or troglodytes or pan troglodytes or gibbon or gibbons or siamang or siamangs or nomascus or symphalangus or chimpanzee or chimpanzees or prosimians or “bush baby” or prosimian or “bush babies” or galagos or galago or pongidae or gorilla or gorillas or pongo or pygmaeus or “pongo pygmaeus” or orangutans or pygmaeus or lemur or lemurs or lemuridae or horse or horses or pongo or equus or cow or calf or bull or chicken or chickens or gallus or quail or bird or birds or quails or poultry or poultries or fowl or fowls or reptile or reptilia or reptiles or snakes or snake or lizard or lizards or alligator or alligators or crocodile or crocodiles or turtle or turtles or amphibian or amphibians or amphibia or frog or frogs or bombina or salientia or toad or toads or “epidalea calamita” or salamander or salamanders or eel or eels or fish or fishes or pisces or catfish or catfishes or siluriformes or arius or heteropneustes or sheatfish or perch or perches or percidae or perca or trout or trouts or char or chars or salvelinus or “fathead minnow” or minnow or cyprinidae or carps or carp or zebrafish or zebrafishes or goldfish or goldfishes or guppy or guppies or chub or chubs or tinca or barbels or barbus or pimephales or promelas or “poecilia reticulata” or mullet or mullets or seahorse or seahorses or “mugil curema” or “atlantic cod” or shark or sharks or catshark or anguilla or salmonid or salmonids or whitefish or whitefishes or salmon or salmons or sole or solea or “sea lamprey” or lamprey or lampreys or pumpkinseed or sunfish or sunfishes or tilapia or tilapias or turbot or turbots or flatfish or flatfishes or sciuridae or squirrel or squirrels or chipmunk or chipmunks or suslik or susliks or vole or voles or lemming or lemmings or muskrat or muskrats or lemmus or otter or otters or marten or martens or martes or weasel or badger or badgers or ermine or mink or minks or sable or sables or gulo or gulos or wolverine or wolverines or minks or mustela or llama or llamas or alpaca or alpacas or camelid or camelids or guanaco or guanacos or chiroptera or chiropteras or bat or bats or fox or foxes or iguana or iguanas or “xenopus laevis” or parakeet or parakeets or parrot or parrots or donkey or donkeys or mule or mules or zebra or zebras or shrew or shrews or bison or bisons or buffalo or buffaloes or deer or deers or bear or bears or panda or pandas or “wild hog” or “wild boar” or fitchew or fitch or beaver or beav or jerboa or jerboas or capybara or capybaras) (Topic) | 8508247 |
| 7 | 5 NOT 6 | 2774 |
| 8 | 7 and Timespan: 2009-01-01 to 2024-04-17 | 2656 |
| 9 | 8 and Science Citation Index Expanded (SCI-EXPANDED) or Social Sciences Citation Index (SSCI) (Web of Science Index) | 2372 |

## Table 1.6 Database: Scopus <1996 to present>

*Search strategy:*

| # ▲ | **Searches** | **Results** |
| --- | --- | --- |
| 1 | ( ( TITLE-ABS-KEY ( ( nutri* W/2 ( quality OR footprint OR food* OR profiling OR density OR score OR index OR adequacy ) ) OR ( food* W/2 ( label* OR suppl* OR consumption OR choice OR environment OR pattern* ) ) OR ( eating W/2 ( indicator OR score* OR impact* ) ) OR ( "feeding behavio*r*" W/2 ( indicator OR score* OR impact* ) ) OR ( "public health" W/2 ( indicator OR score* OR impact* ) ) OR ( health* W/2 ( indicator OR score* OR impact* ) ) ) ) AND ( TITLE-ABS-KEY ( ( sustainab* W/2 ( impact OR assess* OR evaluat* OR indicator? OR health* ) ) OR ( environment* W/2 ( impact OR assess* OR evaluat* OR indicator? OR health* ) ) OR ( climate* W/2 ( impact OR assess* OR evaluat* OR indicator? OR health* ) ) OR ( land* W/2 ( clear* OR "use*" OR usage ) ) OR ( soil W/2 ( clear* OR "use*" OR usage ) ) OR ( water W/2 ( "use*" OR usage OR foot* ) ) OR ( "life cycle" W/2 ( evaluation OR assessment ) ) OR eutrophication OR ( carbon W/2 ( foot* OR ecosystem ) ) OR ( fossil W/2 fuels ) ) ) AND ( TITLE-ABS-KEY ( ( model* OR algorithm* OR metric* OR scor* OR rank* OR framework* OR index OR indices OR tool* OR inventor* OR validation OR "multi-criteria analys*" OR guideline* OR impact* OR optim* ) ) ) AND ( TITLE-ABS-KEY ( ( meal* OR menu* OR recipe* OR diet* ) ) ) ) AND NOT ( animals OR animal OR mice OR mus OR mouse OR murine OR woodmouse OR rats OR rat OR murinae OR muridae OR cottonrat OR cottonrats OR hamster OR hamsters OR cricetinae OR rodentia OR rodent OR rodents OR pigs OR pig OR swine OR swines OR piglets OR piglet OR boar OR boars OR sus AND scrofa OR ferrets OR ferret OR polecat OR polecats OR "mustela putorius" OR "guinea pigs" OR "guinea pig" OR cavia OR callithrix OR marmoset OR marmosets OR cebuella OR hapale OR octodon OR chinchilla OR chinchillas OR gerbillinae OR gerbil OR gerbils OR jird OR jirds OR merione OR meriones OR rabbits OR rabbit OR hares OR hare OR diptera OR flies OR fly OR dipteral OR drosphila OR drosophilidae OR cats OR cat OR carus OR felis OR nematoda OR nematode OR nematoda OR nematode OR nematodes OR sipunculida OR dogs OR dog OR canine OR canines OR canis OR sheep OR sheeps OR mouflon OR mouflons OR ovis OR goats OR goat OR capra OR capras OR rupicapra OR chamois OR haplorhini OR monkey OR monkeys OR anthropoidea OR anthropoids OR saguinus OR tamarin OR tamarins OR leontopithecus OR hominidae OR ape OR apes OR pan OR paniscus OR "pan paniscus" OR bonobo OR bonobos OR troglodytes OR pan AND troglodytes OR gibbon OR gibbons OR siamang OR siamangs OR nomascus OR symphalangus OR chimpanzee OR chimpanzees OR prosimians OR "bush baby" OR prosimian OR "bush babies" OR galagos OR galago OR pongidae OR gorilla OR gorillas OR pongo OR pygmaeus OR "pongo pygmaeus" OR orangutans OR pygmaeus OR lemur OR lemurs OR lemuridae OR horse OR horses OR pongo OR equus OR cow OR calf OR bull OR chicken OR chickens OR gallus OR quail OR bird OR birds OR quails OR poultry OR poultries OR fowl OR fowls OR reptile OR reptilia OR reptiles OR snakes OR snake OR lizard OR lizards OR alligator OR alligators OR crocodile OR crocodiles OR turtle OR turtles OR amphibian OR amphibians OR amphibia OR frog OR frogs OR bombina OR salientia OR toad OR toads OR "epidalea calamita" OR salamander OR salamanders OR eel OR eels OR fish OR fishes OR pisces OR catfish OR catfishes OR siluriformes OR arius OR heteropneustes OR sheatfish OR perch OR perches OR percidae OR perca OR trout OR trouts OR char OR chars OR salvelinus OR "fathead minnow" OR minnow OR cyprinidae OR carps OR carp OR zebrafish OR zebrafishes OR goldfish OR goldfishes OR guppy OR guppies OR chub OR chubs OR tinca OR barbels OR barbus OR pimephales OR promelas OR "poecilia reticulata" OR mullet OR mullets OR seahorse OR seahorses OR "mugil curema" OR "atlantic cod" OR shark OR sharks OR catshark OR anguilla OR salmonid OR salmonids OR whitefish OR whitefishes OR salmon OR salmons OR sole OR solea OR "sea lamprey" OR lamprey OR lampreys OR pumpkinseed OR sunfish OR sunfishes OR tilapia OR tilapias OR turbot OR turbots OR flatfish OR flatfishes OR sciuridae OR squirrel OR squirrels OR chipmunk OR chipmunks OR suslik OR susliks OR vole OR voles OR lemming OR lemmings OR muskrat OR muskrats OR lemmus OR otter OR otters OR marten OR martens OR martes OR weasel OR badger OR badgers OR ermine OR mink OR minks OR sable OR sables OR gulo OR gulos OR wolverine OR wolverines OR minks OR mustela OR llama OR llamas OR alpaca OR alpacas OR camelid OR camelids OR guanaco OR guanacos OR chiroptera OR chiropteras OR bat OR bats OR fox OR foxes OR iguana OR iguanas OR "xenopus laevis" OR parakeet OR parakeets OR parrot OR parrots OR donkey OR donkeys OR mule OR mules OR zebra OR zebras OR shrew OR shrews OR bison OR bisons OR buffalo OR buffaloes OR deer OR deers OR bear OR bears OR panda OR pandas OR "wild hog" OR "wild boar" OR fitchew OR fitch OR beaver OR beav OR jerboa OR jerboas OR capybara OR capybaras ) AND ( LIMIT-TO ( DOCTYPE , "ar" ) ) AND ( LIMIT-TO ( PUBYEAR , 2009 ) OR LIMIT-TO ( PUBYEAR , 2010 ) OR LIMIT-TO ( PUBYEAR , 2011 ) OR LIMIT-TO ( PUBYEAR , 2012 ) OR LIMIT-TO ( PUBYEAR , 2013 ) OR LIMIT-TO ( PUBYEAR , 2014 ) OR LIMIT-TO ( PUBYEAR , 2015 ) OR LIMIT-TO ( PUBYEAR , 2016 ) OR LIMIT-TO ( PUBYEAR , 2017 ) OR LIMIT-TO ( PUBYEAR , 2018 ) OR LIMIT-TO ( PUBYEAR , 2019 ) OR LIMIT-TO ( PUBYEAR , 2020 ) OR LIMIT-TO ( PUBYEAR , 2021 ) OR LIMIT-TO ( PUBYEAR , 2022 ) OR LIMIT-TO ( PUBYEAR , 2023 ) OR LIMIT-TO ( PUBYEAR , 2024 ) ) | 2740 |
