# Supplementary material for: Food-based indices for the assessment of nutritive value and environmental impact of meals and diets: A systematic review protocol
Source: PLoS One. 2024 Dec 20;19(12):e0315894. doi: 10.1371/journal.pone.0315894 (PMC11661603; doi:10.1371/journal.pone.0315894)
Supplement: Attachment — Submitted filename: Response to Reviewers_ELT_PO.docx [file pone.0315894.s003.docx]

**Response to Reviewers and Editor**

**Note to editor:** Thank you for organising the reviewers, their comments have been helpful and informative. We believe that we have suitably addressed their concerns through changes to the manuscript and further explanations in the response below, resulting in a better paper overall.

**Note to reviewers:** We would like to thank the reviewers for taking the time to carefully review the manuscript and for their insightful feedback.

| **Reviewers comments** | **Author’s reply** | **Revision** |
| --- | --- | --- |
| Authors have designed a study protocol to investigate Food-based indices for the assessment of nutritive values and environmental impacts of meals and diets. Results provide very valuable information to public and governmental sectors to support healthy and sustainable meal choices and diets. The MS was well designed, however it needs revision before being accepted for publication. |  |  |
| The manuscript presents clearly identified and justified research questions, and the methods provide sufficient detail to allow for reproduction.  Supporting information on PRISMA-P check list and search strings details for each database are accessible. The protocol can be accepted for publication. |  |  |
| 1. Please ensure that your manuscript meets PLOS ONE's style requirements, including those for file naming. | Following your comments, the PLOS ONE style templates were used to double check and ensure the manuscript (including titles, tables, table citations, references) and supporting information were formatted correctly. In addition, the S1 File_PRISMA-P Checklist’ was updated to reflect the line numbering of the revised manuscript.  One sub-heading was found that did not comply to the correct formatting, which has now been amended, and the supplementary files have now been updated to reflect the correct file naming format.  Finally, the acknowledgments were moved further up in the MS to follow-on directly from the conclusion and the reference list was moved to sit ahead of the supporting information, as per the PLOS ONE style templates. | L193 amended to level 3 heading.  File name amended from ‘S1 File. PRISMA-P Checklist’ and ‘S2 File. Search string details for each database’ to ‘S1 File_PRISMA-P Checklist’ and ‘S2 File_Search string details for each database’.  Acknowledgements moved to L354.  References moved to L375. |
| 2. As required by our policy on Data Availability, please ensure your manuscript or supplementary information includes the following:   A numbered table of all studies identified in the literature search, including those that were excluded from the analyses.  For every excluded study, the table should list the reason(s) for exclusion.  If any of the included studies are unpublished, include a link (URL) to the primary source or detailed information about how the content can be accessed.  A table of all data extracted from the primary research sources for the systematic review and/or meta-analysis. The table must include the following information for each study:  Name of data extractors and date of data extraction  Confirmation that the study was eligible to be included in the review.  All data extracted from each study for the reported systematic review and/or meta-analysis that would be needed to replicate your analyses.  If data or supporting information were obtained from another source (e.g. correspondence with the author of the original research article), please provide the source of data and dates on which the data/information were obtained by your research group.  If applicable for your analysis, a table showing the completed risk of bias and quality/certainty assessments for each study or outcome.  Please ensure this is provided for each domain or parameter assessed. For example, if you used the Cochrane risk-of-bias tool for randomized trials, provide answers to each of the signalling questions for each study. If you used GRADE to assess certainty of evidence, provide judgements about each of the quality of evidence factor. This should be provided for each outcome.  An explanation of how missing data were handled.  This information can be included in the main text, supplementary information, or relevant data repository. Please note that providing these underlying data is a requirement for publication in this journal, and if these data are not provided your manuscript might be rejected. | We thank the reviewer for their enquiry into the data availability of this review. We agree that a manuscript for a systematic review requires  a numbered table of all studies identified in the literature search, including those that were excluded from the analyses. However, this manuscript is submitted as a systematic review protocol, and therefore data collection had not been completed at the time of manuscript submission. Therefore, it is not possible to include the above, however, this data will be included in the supplementary material of the systematic review manuscript.. | Now starting on line 291 now reads:  *“All extracted data will be included in the systematic review manuscript.”.* |
| 3. Please review your reference list to ensure that it is complete and correct. If you have cited papers that have been retracted, please include the rationale for doing so in the manuscript text, or remove these references and replace them with relevant current references. Any changes to the reference list should be mentioned in the rebuttal letter that accompanies your revised manuscript. If you need to cite a retracted article, indicate the article’s retracted status in the References list and also include a citation and full reference for the retraction notice. | In response to the reviewers request the full reference list was reviewed to identify any retracted papers. None of which were identified.  In addition, the referencing format of all references included in the manuscript were double checked against the PLOS One and some minor amendments were made which included the listing of organisation names in full. | References amended were made to the below references where organisation names were written out in full.   - Reference 4 (L360) - Reference 6 (L366) - Reference 8 (L374) - Reference 16 (L402) - Reference 17 (L406) |
| 4. Abstract should be shortened. It is too long for the necessity and strength of the study. Please fix it in limited number of words. I believe it to be possible with max. 350 words. | Following your comment, we acknowledge and agree that the abstract needed to be shortened. Background information has been shortened to focus on the key information that readers need to know to understand the background for the protocol to follow. The detail in the methodological section was kept, but where possible wording was made more succinct. Wording was additionally, condensed in the conclusion of the abstract but the key message around providing valuable information to public and governmental sectors to support healthy and sustainable meal choices and diets remains. The abstract word count is now 334 words. | Now starting in line 21 it reads:  *“Current food production and consumption practices are impacting both human and planetary health. Though these challenges are multifaceted, shifting to healthy dietary choices from sustainable food systems is one solution. Food-based labelling is a common public-health strategy aimed at influencing consumption practices, primarily displaying front-of-pack nutrition labelling to encourage healthier choices. Due to the dual impact of food on human and planetary health there is a growing need to additionally include environmental impact information. However, this potentially conflicting information could overwhelm consumers, necessitating a simplified approach that combines nutritive and environmental values.. Previous work has identified existing models, termed sustainable food profiling models, for labelling of individual food products based on their environmental impacts and nutritive value. Foods are rarely eaten in isolation and are often consumed as composite meals, which aggregate to diets. Therefore, it is important to identify indices that exist to assess or rank meals and diets according to their nutritional and environmental impacts.*  *Following the Preferred Reporting Items for Systematic Review and Meta-Analysis Protocols (PRISMA-P) 2015 guidelines, our systematic review protocol was registered with the International Prospective Register of Systematic Reviews (PROSPERO) on 01 May 2024 (PROSPERO registration ID = CRD42024537149).*  *In the present protocol the methodology to review identify existing food-based indices for the assessment of nutritive value and environmental impact of meals and diets and their intended public health purpose is described. Our primary research questions are: (i) what food-based indices assessing nutritive value and environmental impact exist for classification or ranking of meals or diets? and (ii) what are the methods used to create, and the key characteristics of, these indices?.*  *The results are expected to increase understanding of, and highlight the variation in, the creation of combined measures for the assessment of nutritive value and environmental impact for meals and diets. Additionally, findings can be used to inform researchers, business, and policy actors on future approaches for labelling foods, meals, or diets in a way that supports healthy and sustainable meal choices and diets.”* |
| 5. Re-wording & grammatical comments:   L28: to express L28: and should not be Italic. L28: values L37: impacts L39: comma after guidelines L42: in the present study as a substitute for here L42: methodology to review food-based indices determined for …. L68: comma after land  L111: These as a substitute for this (Many reason are aforementioned to stress strength of the present study).  L150 – 151: Please omit “Search results will be downloaded and catalogued in EndNote version 21.3.”  L157-158: please omit (science and social science index)  L195: will be used  L198: It may be better to use This criteria as a substitute for this review   L208: This criteria will review … | We agree with all reviewer comments on the suggested wording and grammatical comments and therefore these have all been amended in the manuscript. | L32 – “to express” removed.  L32 - Italics removed.  L32 – “Value” amended to “values” added.  L41 – “environmental impact” amended to “environmental impacts”.  L43 – comma added.  L46 – “In the present study” used to replace “the protocol described here”  L47 – “to review food-based indices” used to replace “for a systematic review to explore what”  L73 – comma added.  L16 – “This” amended to “these”.  L159 - “Search results will be downloaded and catalogued in EndNote version 21.3.” now omitted.  L167 – “(science and social index)” now omitted.  L207 – “were” amended to “will be”.  L210 – “This criteria” used to replace “This review”.  L220- ”This criteria will review” used to replace “this study”. |
| 6. L49-53: Outcomes could be shorten. | We agree with you that outcomes expected from the systematic review could be shortened. To condense this, duplicating and unnecessary words were removed. | Now starting in line 56 it reads:  *“Additionally, findings can be used to inform researchers, business, and policy actors on future approaches for labelling foods, meals, or diets in a way that supports healthy and sustainable meal choices and diets.”* |
| 7. L76: I think the best is to use environmental impacts through the MS. It is better than environmental impact. >>> L87, L89, L91, L112, L205 and more. | Environmental impact has been amended throughout the manuscript to “environmental impacts” where it is appropriate. | Environmental impact amended to environmental impacts throughout manuscript in L34, 41, 92, 94, 96, 103, 117, 134, 140, 143, 216, 217, 221, 222, 224, 229, 232, 234, 239, 244, 269 and 270. |
| 8. L98: Similar to former comment, I think nutritive values  could be considered as a substitute for nutritive value  through the MS. >>> L112, L205. | We carefully considered amending the term “nutritive value” to “nutritive values”, however we believe “nutritive value” to be the most appropriate term to use. |  |
| 9. L116 – 125: Please re-structure. Use of bullets is not common. | We agree that re-structuring the layout of the PICO framework is more appropriate for a journal article and is now formatted as free-flowing text. | Now starting in line 122 it reads:  *“There is no restriction on population as nutritive and environmental combined indices for the assessment or ranking of meals or diets should be applicable and suitable for all population groups. Nutritive value and environmental impact indices for assessment or ranking of meals and diets are considered the intervention with no comparator. the outcomes considered are the number of combined indices including a nutrition score dimension and an environmental impact dimension for the assessment or ranking of meals or diets, methodology of combination, key characteristics, and public health purpose.”* |
| 10. L126 – 130: Please re-structure. You may use: The present study was conducted to understand What food-based indices ….. and what are the methods used | We agree that re-structuring the layout of the primary and secondary aims is more appropriate for a journal article and is now formatted as free-flowing text. | Now starting in line 129 it reads:  *“The present study protocol is described to understand what food-based indices assessing nutritive value and environmental impacts exist for classification or ranking of meals or diets? And what are the methods used to create, and the key characteristics of, these indices?”* |
| 11. L144: formed in a checklist (Supplementary material – File #1) [18]. | The suggested wording of referring to the supplementary material, including file number and the exact appendix has been implemented throughout the manuscript. | Now starting in line 151 it reads:  *“The 17 items that are considered necessary to report a systematic review protocol are formed in a checklist (Supplementary material – File #1) [18].”* |
| 12. L155: Supplementary material (File #2, appendix S1). Please use this formulation for citation of the supplementary material in the text. >>> L171 | See above response. | Now starting in line 163 it reads:  *“Keywords from these relevant papers were identified and grouped into themes based on the PICO elements of this systematic review (Supplementary material – File #2, appendix S1).”*  Now starting in line 181 it now reads:  *“The search string details for each database can be found in the Supplementary material – File #2, appendix S2.”* |
| 13. L161: Rӧӧs et al. [21], Seconda et al. [22] and van Dooren et al. [23]. | We agree with the proposed layout of referencing and have amended to the reviewers suggestions to improve citation clarity. | Now starting in line 170 it reads:  *“Key papers identified as work of Rӧӧs et al. (2015) [21], Seconda et al. (2019) [22] and van Dooren et al. (2014) [23].”* |
| 14. L172: Search string for bibliographic databases | We agree with the removal of the unnecessary words “to be used”. | Now starting in line 183 it now reads:  *“Table 1. Search string to be used for bibliographic databases (four components will be combined with the Boolean operator AND before combination with the Boolean operator ‘NOT’ for the fifth component).”* |
| 15. L180, L190, L240: reviewers? It may better to use “authors”. I am wondering reviewers are incorporated into study design | The manuscript has been revised to use the term “authors” as a replacement for “reviewers” to increase the clarity on who carried out the screening strategy and the data extraction. | Now starting in line 189 it reads:  *“Article data will be exported from EndNote, a reference management software package, and imported into Ryyan, a web-based systematic review software, for a two-staged screening strategy by two independent, blinded reviewers authors.”*  Now starting in line 201 it reads:  *“Where there is uncertainty, the article will be marked for review by a third author.”*  Now starting in line 202 it reads:  *“Ryyan provides the number of conflicts between authors, from this a decimal (amount of agreements / number of papers screened) can be calculated”.* |
| 16. L282: Data synthesis : Please describe this section in details. What will be done with data including statistical tests that may be useful to answer the questions (hypotheses) | Due to the qualitative nature of this work statistical tests will not be carried out and hence were not discussed in the original manuscript. Two sentences were added to increase understanding of how the extracted data will be synthesised and presented. | Now starting on like 296 it reads:  *“Statistical analysis will not be carried out due to the qualitative nature of data that will be collected and therefore conducting a narrative synthesis will allow authors to understand how the interventions work, why and for who, enabling exploration of relationships in the data and allowing for strength of evidence assessment in order to draw evidence based conclusions [25]. Counts will be used to report the number of identified indices and a heterogenous table will be used to report the general information. Summary and matrix tables will be used to present extracted data relating to the intervention and outcomes with supporting narrative synthesis to enable comparisons between the indices included in the review, and to answer the primary and secondary research questions.”* |
| 17. L294 – 300: Authors may stress the impacts of nutritional values on health. For instance there are frequent studies highlighting the impacts of high-fat-diets on prevalence of diabetes, cognitive disorders and …. | After discussion we felt that addition of reviewer suggestion would bias the conclusion in favour of the nutritional impacts of food consumption, where the systematic review protocol aims to assess both the nutritive and environmental impacts. |  |
| 18. L301 – 304: This is very important limitation, please discuss in detail. It might be used as a criteria to determine the eligibility of a study to be incorporated into Meta-analysis. For instance, if sample size for determination of nutritional values are well enough, if grouping of foods for determination f nutritional values was rational, etc | We agree with the author that this is an important limitation in this work, however, there is no data that is planned to be collected that would be appropriate for a meta-analysis, additionally, sample size is not expected to be featured in the studies we are expecting to capture in this systematic review and hence expansion of this point for these purposes would not be necessary. |  |
| 19. Have the authors described where all data underlying the findings will be made available when the study is complete?  The [PLOS Data policy](https://eur02.safelinks.protection.outlook.com/?url=https%3A%2F%2Ftrack.editorialmanager.com%2FCL0%2Fhttps%3A%252F%252Fjournals.plos.org%252Fplosone%252Fs%252Fmaterials-and-software-sharing%2F1%2F010f019355c40734-d790298f-2674-42cd-9cdd-75058917b6b7-000000%2FHfdNuVSmAW-9LvfbRRDFWd9cUnJW2cVdbx2eCG267rI%3D186&data=05%7C02%7Cethomas08%40qub.ac.uk%7Cd6ef7c7e0b8e4dc7ea3508dd0b3c322f%7Ceaab77eab4a549e3a1e8d6dd23a1f286%7C0%7C0%7C638679076010904560%7CUnknown%7CTWFpbGZsb3d8eyJFbXB0eU1hcGkiOnRydWUsIlYiOiIwLjAuMDAwMCIsIlAiOiJXaW4zMiIsIkFOIjoiTWFpbCIsIldUIjoyfQ%3D%3D%7C0%7C%7C%7C&sdata=TqocUu%2F1jYZX7GDQuAFnG9TrcpNp47f3WL%2FOnSsHf2c%3D&reserved=0) requires authors to make all data underlying the findings described in their manuscript fully available without restriction, with rare exception, at the time of publication. The data should be provided as part of the manuscript or its supporting information, or deposited to a public repository. For example, in addition to summary statistics, the data points behind means, medians and variance measures should be available. If there are restrictions on publicly sharing data—e.g. participant privacy or use of data from a third party—those must be specified.   Reviewer #1: No | We welcome the reviewer comments regarding transparency and accessibility to data included in manuscripts, however, we refer back to the author response to comment 3, whereby there are no included data and no presented findings in a protocol manuscript.  A sentence was asses to convey that all data will be reported and available in the systematic review manuscript once data collection is complete in the data extraction sub-section. | Now starting in line 292 it reads:  *“All extracted data will be included in the systematic review manuscript”* |
